# A Smartphone Crowdsensing System Enabling Environmental Crowdsourcing for Municipality Resource Allocation with LSTM Stochastic Prediction

**DOI:** 10.3390/s20143966

**Published:** 2020-07-16

**Authors:** Theodoros Anagnostopoulos, Theodoros Xanthopoulos, Yannis Psaromiligkos

**Affiliations:** 1 DigiT.DSS.Lab, Department of Business Administration, University of West Attica, Thivon 250, Egaleo 122 44 Athens, Greece; xanthopoulos@uniwa.gr (T.X.); yannis.psaromiligkos@uniwa.gr (Y.P.); 2 Department of Infocommunication Technologies, ITMO University, Kronverkskiy Prospect, 49, 197101 St. Petersburg, Russia

**Keywords:** smartphone crowdsensing, environmental crowdsourcing, edge mobile applications, stochastic prediction, LSTM, department resource allocation, municipality

## Abstract

Resource allocation of the availability of certain departments for dealing with emergency recovery is of high importance in municipalities. Efficient planning for facing possible disasters in the coverage area of a municipality provides reassurance for citizens. Citizens can assist with such malfunctions by acting as human sensors at the edge of an infrastructure to provide instant feedback to the appropriate departments fixing the problems. However, municipalities have limited department resources to handle upcoming emergency events. In this study, we propose a smartphone crowdsensing system that is based on citizens’ reactions as human sensors at the edge of a municipality infrastructure to supplement malfunctions exploiting environmental crowdsourcing location-allocation capabilities. A long short-term memory (LSTM) neural network is incorporated to learn the occurrence of such emergencies. The LSTM is able to stochastically predict future emergency situations, acting as an early warning component of the system. Such a mechanism may be used to provide adequate department resource allocation to treat future emergencies.

## 1. Introduction

Nowadays, human habitation is heading in the direction of smart cities—or cities 2.0—made possible due to technological advancements based on the Internet of things (IoT) [1,2,3]. We studied the municipality of Papagos–Cholargos, a smart city located in Athens, Greece. Here, the municipality of Papagos–Cholargos has developed a technical infrastructure that enable citizens to act as human sensors by exploiting their smartphones to report malfunctions in the municipality infrastructure. Using the Citify software application, problematic situations in the municipality are annotated and submitted to the system for further processing empowered with crowdsourcing and crowdsensing technology [4,5]. When a report arrives to the municipality control center the system allocates certain department to serve the problem. For better handling problematic situations, the municipality is further divided in to sections; section of Papagos and section of Cholargos. Since incidents are served by a certain number of departments with limited resources, the early planning and allocation of a department’s resources before the incident emerges is of crucial significance. To handle such situations, we used an inference engine model that is based on a long short-term memory (LSTM) neural network to learn stochastically from the past examples of an incidence occurrence. Based on the LSTM classification learning, provided by Weka machine-learning workbench [6], the proposed system is able to predict a future emergence of a similar event thus allocates efficiently a municipality department resource before the problem takes place in the future.

The motivation of this study is to solve a department resource allocation problem that actually exists at the municipality of Papagos–Cholargos under the assumption that the municipality needs an emergency manual that will enable the proper function of the municipality administrative tasks through the mapping of upcoming events to certain departments within the period of a year. The challenging issue it that such emergency manual does not following a straightforward approach. Instead of using hardcoded instructions to create such a manual we used an LSTM inference model to define the relationships of such a mapping and create it based on stochastic data. Specifically, as it is explained in the definition of the problem the relationship between an event and a department is that the event must be served by an available department. Please note that the jurisdiction of each department is to handle many other municipality administrative tasks as well as the discussed events presented in the study. Being able to define the workload of a certain department during the year for serving certain events, this gives the municipality the opportunity to use other departments to handle other administrative tasks that is actually a resource allocation issue. Specifically, there is not an a priori knowledge about how many departments are required during the year to serve such events out of the total amount of administrative tasks that a municipality may require at any given time. The reviewer is correct about the meaning of a well-designed emergency manual. What we produce as an outcome of the proposed research model is how we currently handle certain methodologies (i.e., crowdsensing, crowdsourcing, human sensors, inference LSTM model). A well-designed emergency manual for the municipality of Papagos-Cholargos is actually needed for the proper functioning of the municipality. Please also note current outputs do not help users to find corresponding departments themselves. Users give stochastic input to the system as human sensors, and they do not choose the appropriate municipality department to treat the event. Currently, users do not intervene in the municipality operation and department invocation resource allocation. This is a task that the system does due to the inferred emergency manual that has been developed based on prior knowledge. Such knowledge exists in the LSTM model and with the enhancement of current observed events provided by users is able to perform an intelligent resource allocation prediction rather that a hardcoded mapping.

Using citizens as human sensors and artificial intelligence models to predict upcoming situations also uses crowdsourcing in the literature for annotating problematic situations in municipalities. Support of massive data requirements observed by the crowdsourcing process that inputs supervised machine-learning algorithms to match certain volunteer contributors to appropriate tasks are discussed in [7]. A crowdsourcing (probably approximately correct) PAC learning is used to take unlabeled data points and input them to a majority voting ensemble classification model to assign the appropriate labels in [8]. Quality of human sensors with regards to the dimension of efficiently annotate emerged problems in the municipality can be observed in [9]. Filtering data occurred by crowdsourcing process is also a significant issue when citizens report problems with their smartphones as described in [10]. A crowdsourcing system that includes labeling of an image collection that uses two sets of images to identify citizens acting as human sensors, is analyzed in [11]. Crowdsourcing can also be used as a viable platform for conducting different types of data evaluation to purify content quality, is discussed in [12]. Citizen crowdsensing form the smart cities point of view is discussed in [13] where research covers the areas of municipality platforms for environmental sensing and problem reporting. Smartphone crowdsensing is also treated as a road sustainability problem in [14] that is treated under the exploitability of municipality data publicly available.

Another technology aims to treat emerged problems in the municipality infrastructure with the use of smartphones is crowdsensing. In such environment, citizens are transformed to human sensors that can be aware, track and annotate with image, video and text or voice a problematic issue in the municipality coverage area and transmit such data to the appropriate inference engine model for further processing. Specifically, in [15] it is presented a system that enhances data located in social networks, green applications, municipality environmental monitoring and smart transportation systems. Such data are further processed by the use of mobile technology to provide environmental protection. Human sensing with smartphone technology towards volunteer spatiotemporal context with application in municipality geographic area is also discussed in [16]. Specifically, smartphones are used to enable the transformation of citizens to human sensors to sense the municipality area and track unusual behavior. Mobile crowdsensing is a promising paradigm for cross-space and large-scale sensing as discussed in [17]. Mobile crowdsensing extends the area of participatory sensing by transforming both participatory sensory data provided from mobile devices—as well as user contributed data sources from mobile social networking services—focusing on the collaboration of machine and human intelligence in crowdsensing and computing processes. A hybrid crowdsensing system that incorporates social media-based paradigm, aims at combining the strengths of both participatory and opportunistic crowdsensing, as described by the authors in [18]. Specifically, the system is able to demonstrate its feasibility and usefulness by enabling users to contribute contact and provide additional information following a participatory and opportunistic approach.

Human sensing through crowdsourcing and crowdsensing is further used in our concept for disaster emergency planning and early recovery of unpleasant events in the municipality. In such situations the first thing needs treatment is the location-allocation of the problematic event and it follows the disaster recovery. Specifically, in [19] it is defined the location allocation problem in the context of municipality spatial area with regards to crowdsourcing and crowdsensing technologies, while in [20] it is presented a system that provides a possibilistic programming approach to treat location allocation of an event with the incorporation of fuzzy decision-making techniques. Subsequently, in [21] it is presented a location allocation approach that focuses on a multiple criteria decision-making system to treat facility location problems. A disaster risk management framework for disaster recovery and efficient inventory planning is proposed in [22]. Such system uses newsvendors’ variants that exploits demand uncertainty to treat an emerged event. A robust humanitarian decision support system for disaster recovery and planning is presented in [23]. Such a system exploits robust humanitarian facility location within the coverage area of municipality. Monitoring user trajectories on a daily basis is an essential requirement to provide advanced mobile services using crowdsensing technology. In [24], authors propose a mobility prediction model that is able to provide contextual information about a user’s mobility in a smart city by detecting efficient sensing schedules. A systematic study of the coverage and scaling features of place and temporal centric crowdsensing is analyzed in [25]. The analyses of certain data sources based on place and temporal centric crowdsensing exploits the relationship between user population in a smart city and the characterization of the collected data with regards to coverage and privacy concerns.

Resource allocation, management and planning are significant parameters for the effective operation of a municipality. As described in [26], crowdsourcing can be used as a human-centric tool to track and report malfunctions in a municipality that can concretely support an urban process for efficient emergency management in case of disaster. Such technology can be combined with participatory crowdsensing to provide challenges and opportunities to the design of further infrastructure systems for the municipality, provided in [27]. In addition, it is able for the municipality to benefit and develop a wellbeing environment for its citizens by adopting certain enterprise resource allocation and planning systems, analyzed in [28]. A feasibility study exploiting crowdsourcing and crowdsensing technologies proved that smart cities’ wellbeing is getting better with the incorporation of monitoring technology that is applied on municipality resources, as described in [29]. Municipalities can be supported by intelligent decision-making systems that analyze historic data to provide viable solutions for their citizen wellbeing, as discussed in [30]. Quality control in crowdsourcing systems is discussed in [31], where the authors propose a framework that characterizes various dimensions of quality control in such systems, identify open issues and proposes future research trends. A crowdsensing system for dealing with disaster recovery and providing humanitarian assistant is discussed in [32]. Specifically, the system handles emergency situations that require leveraging situation aware data sources. A set of mechanisms are presented that are used for connecting and utilizing crowdsourcing and crowdsensing data within a smart city to provide disaster recovery and planning. Authors in [33] propose a system that uses crowdsensing to discover an emergency and subsequently produce recovery planning. The problem is treated as a sequential change point detection situation, where smart emergency management processes are attracting increasing attention due to their capability to potentially save citizen lives.

Crowdsourcing to smartphones approach, as presented in [34], assesses pervasive smartphones to collect and analyze data in a more effective way than was previously possible. The proposed system incorporates advanced incentive mechanisms that can attract greater user participation through a well-designed mobile phone sensing platform. The authors in [35], propose a system that enables privacy and preserves incentives for mobile crowdsensing systems. The proposed approach is based on the single-minded reverse combinatorial auction that is able to provide a guaranteed approximation ratio to face upcoming problems. An honest incentivizing mobile data crowdsourcing system is proposed in [36]. Such approach incorporates truthful crowdsourcing mechanisms to incentivize strategic workers to truthfully report their progress. An incentivizing multi-requester mobile crowdsensing system is proposed in [37]. In this approach, an incentive mechanism is presented that is based on double auction and is able to evaluate the participation of both information requesters and workers. The authors introduce CENTURION that is an integrated framework for multi requester mobile crowdsensing systems to generate highly accurate aggregated results on system performance.

Current research in contemporary technology for municipalities exploits certain techniques and technologies. There are studies that focus of either crowdsourcing or crowdsensing technology to monitor and contribute to the efficient operation of municipality resources. Such operations can handle emergency situations and improve citizen wellbeing. However, in our approach we are treating citizens as human sensors to contribute to municipality effectiveness by tracking and annotating problematic situations. In addition, we learn from historic past data regarding the areas and the time where certain problems occurred. Each problem is assigned to a specific department to get solved, where the municipality of Papagos–Cholargos is divided in two sections for better treat each emerged problem. Concretely, a LSTM model is used to be trained on these data and be able to predict when such a new unseen upcoming problem will occur in the future. The adopted artificial intelligence-based system enables the municipality to perform efficient resource allocation and disaster planning proactively in case of emergency that optimizes citizens wellbeing.

## 2. Materials and Methods

Municipality department resource allocation exploits data gathered by the municipality of Papagos–Cholargos in Athens, Greece. Specifically, such a municipality is separated in two sections, namely section of Papagos and section of Cholargos that are neighbor municipality sections. Certain system is built to serve the resource allocation needs of both sections and the whole municipality.

### 2.1. System Overview

**Figure 1:**
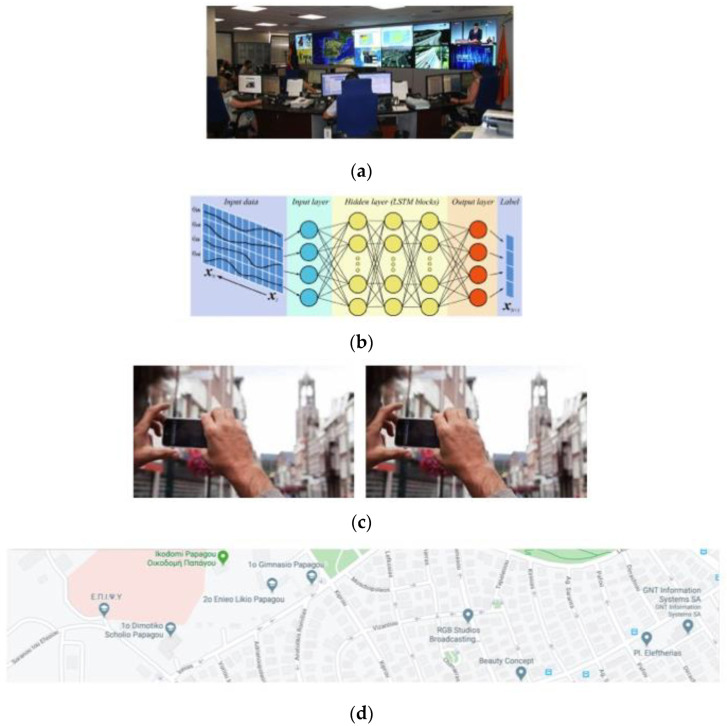
Overview of the four layers of the proposed system. (**a**) Municipality headquarters layer; (**b**) inference engine model layer; (**c**) smartphone crowdsensing layer; and (**d**) environmental crowdsourcing municipality layer.

Four discrete layers compose the proposed system. From bottom to top, the first layer contains the environmental crowdsourcing municipality area that is the physical area of the municipality extended in both sections, where citizens can be located in the coverage area and perform crowdsourcing processes. This layer is composed of the municipality map focusing on the citizens home locations, as well as the road addresses at the edge of the system. The second layer depicts the smartphone crowdsensing process that is decomposed to the citizen population acting as human sensors. Each human sensor has a smartphone application that is used to track and annotate any problem that may arise in the first layer. In the case a problem occurs at a certain road address, a human sensor takes a photo of the problem and annotates the situation with a text message. Such information is passing to the next layer. The third layer is composed of the inference engine model that runs on the cloud and is capable of taking the annotated problem from the previous layer, processing, it and assigning the problem to specific department resources. Resource allocation is feasible due to third-layer processing. In addition, the model is able to learn from historical data (i.e., past annotated problems assigned to certain department) and uses such knowledge to train a long short-term memory (LSTM) classifier. When the LSTM is trained, it is further used to make predictions and propose solutions for assigning certain departments to face specific problems, thus performing department resource allocation. The last layer contains the municipality headquarters that is responsible to monitor the municipality coverage area and the processes performed by the previous layers. In this layer, a decision is made based on the proposed department resource allocation solution for a certain problem developed by the inference engine model. When a decision is made, the municipality workers solve the problem following a certain recovery process. The proposed system overview is presented in Figure 1.

### 2.2. Citify Software Application Interfaces

We have implemented the Citify software application to handle the emergence of a certain problem in the municipality of Papagos–Cholargos. Citify has two graphic user interfaces (GUI) related to the two main actors of the system: (a) human sensor interface and (b) municipality headquarters’ monitoring interface. The smartphone application GUI is used for the human sensor interface while the web application interface is used for the needs of the municipality headquarters.

#### 2.2.1. Smartphone Application Interface

**Figure 2:**
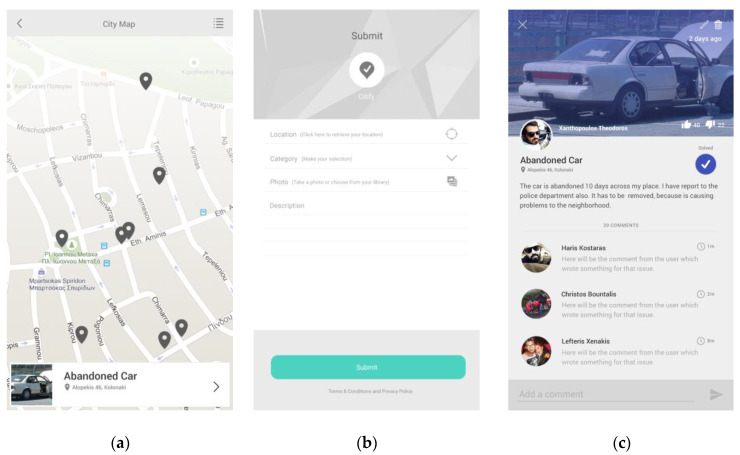
Three steps required to face the problem: (**a**) track the problem on the municipality map road address; (**b**) citizen annotates the problem and submits it to the system; (**c**) system informs citizen that the problem has been fixed.

Upon the emergence of problem in the municipality coverage area, a human sensor that acts asynchronously compared to other crowdsourcing human sensors uses crowdsensing technology to track, annotate and submit to the inference engine model. This process runs at the edge of the system and is invoked for each problem that may arise. It has three steps. First, the problem is tracked by exact road address; then it is annotated with a photo and a text message. Then, the problem is submitted to the system and is further processed. When the municipality workers fix the problem according to the exact recovery plan, the system informs the citizen through the smartphone application interface that the problem has been solved. This process is depicted in Figure 2.

#### 2.2.2. Web Application Interface

**Figure 3:**
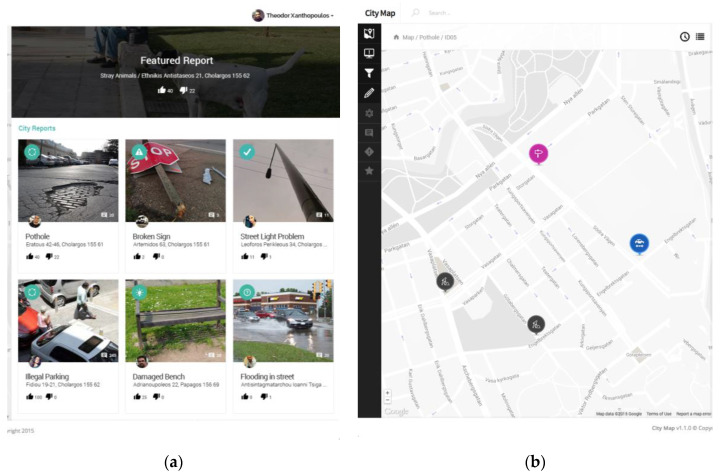
Web application: (**a**) left is observed the municipality map with road addresses; (**b**) right are observed the emerged problems that should be fixed by the system.

The municipality headquarters has the overall responsibility to assure that everything in the map coverage area is monitored. In addition, the headquarters is responsible to make correct decisions and fix emerging problems. A web application supports the municipality efforts by providing all available information to the appropriate personnel. Such an application visualizes the upcoming problems that have been reported by citizens through the crowdsensing smartphone application, where citizens are acting within the municipality environmental crowdsourcing coverage area. Specifically, each reported problem is assigned to a certain municipality road address, while the progress of the process is also presented. When an incident is fixed, then the web application annotates it in the map and sends a notification message to the human sensor’s smartphone application interface. The web application interface is used to serve the municipality of Papagos–Cholargos is presented in Figure 3.

### 2.3. System Architecture

**Figure 4:**
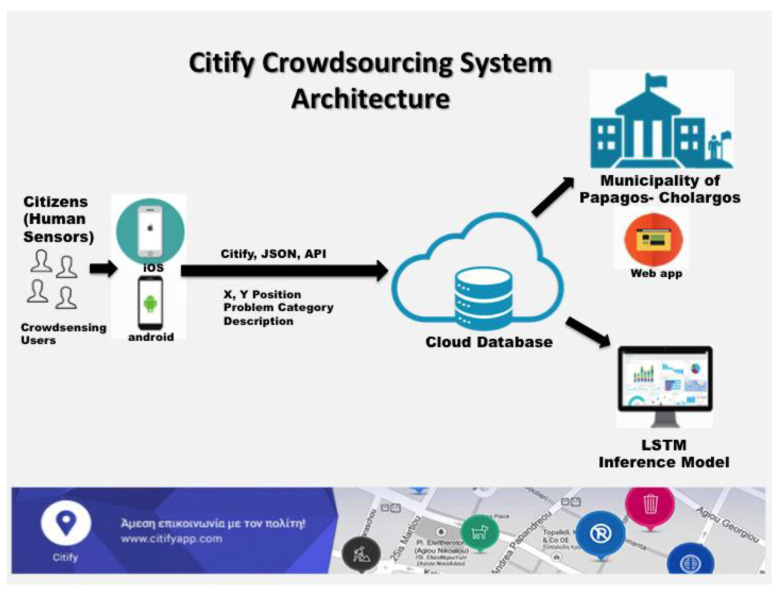
Proposed crowdsourcing system architecture.

The proposed crowdsourcing system architecture is shown in Figure 4. Specifically, we treat citizens as human sensors, where they are acting as crowdsensing users to track and annotate an event within the municipality coverage area. Such tracking is achieved by sending reports to the system via the Citify application. Annotation is used to provide spatial positional data, event category and appropriate description (i.e., image or audio data) to the system. Image and audio data are not further used by the system; instead, there are used only for annotation. On the contrary, spatial location data and event category is used to feed cloud database through the JSON Citify API. LSTM inference model that uses stochastic data and new unseen data observed by current tracking and annotation to provide predictions for exact department availability to provide service of the event. The municipality of Papagos–Cholargos web application evaluates such prediction.

**Figure 5:**
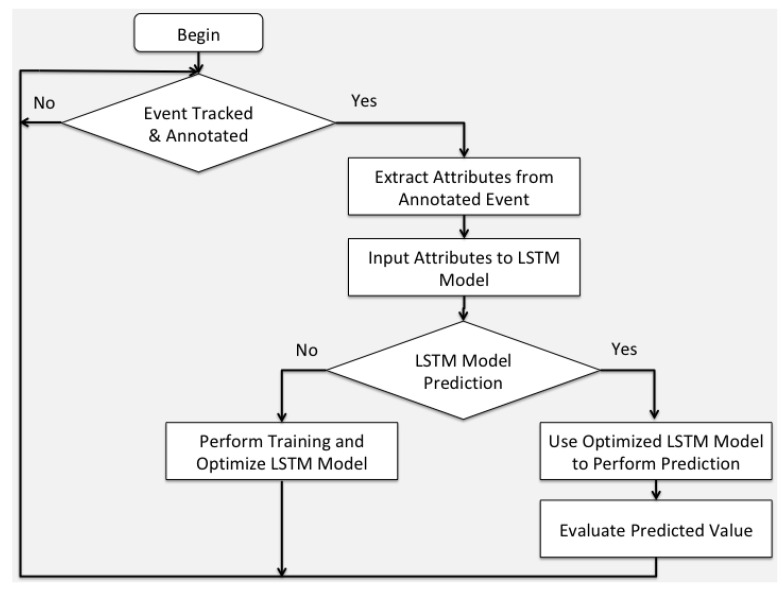
Data flow of the proposed system.

The data flow of the proposed system architecture is presented in Figure 5. On the emergence of a tracked and annotated event, the system records the extract attributes from the message sent by the human sensor. Such attributes are input into the LSTM inference model, and the system decides if it is a training or a prediction invocation call. In the case it is a training invocation, a system training is performed that outputs an optimized LSTM model. In the opposite case of prediction invocation, the system uses optimized the LSTM model to perform a prediction that is then evaluated by assessing system efficiency.

### 2.4. System Modeling

#### 2.4.1. System Model for Municipality and Municipality Sections

The municipality of Papagos–Cholargos is divided in two sections—the Papagos section and the Cholargos section. Each section is further divided to sectors that are used for better handling of an upcoming problem. In addition, the inference engine model is composed by an LSTM classifier that is trained based on certain datasets. Data model describing the structure of each dataset is depending on the classification model that is also based on the examined map coverage area either of the municipality or the municipality section. Specifically, crowdsourcing processes occurring in the municipality environmental area enable crowdsensing layer to feed the LSTM classifier with certain predictive attributes that are namely road address, problem description, month of occurrence and municipality section’s sector that the problem occurred, while the class attribute is the assigned department to handle the upcoming problem. In case of Papagos section the values of the predictive and class attributes are depicted in Table 1, while for Cholargos section the values of the dataset are presented in Table 2. Note that when we treat the municipality of Papagos–Cholargos as a whole entity we merge these two datasets since they have the same structure as shown in Table 3. In addition, to focus on which part of the Papagos–Cholargos section, we provide a prediction to add the appropriate section as a predictive attribute. In Table 1, we can observe that Papagos section contains 10,000 road addresses and 20 sectors for handling local problems. In Table 2, we can see that Cholargos section has 12,000 road addresses and 24 local sectors. In Table 3 we can see that municipality of Papagos–Cholargos has 2 sections (i.e., Papagos and Cholargos), 22,000 road addresses and 44 overall sectors in both sections. Both municipality sections can handle a variety of 15 problems. Such problem value descriptions are described in Table 4. Number of months are 12 that means that the system serves municipality needs for the whole year of operation. The cardinality of the class attribute, i.e., departments is 10. Note that each department can serve simultaneously Papagos section and Cholargos section (i.e., the whole municipality of Papagos–Cholargos) according to upcoming needs for problem assistance within a year. In Table 1, Table 2, Table 3 and Table 4, we present the features (i.e., attributes) with regards to certain types and values obtained for each of them, as extracted for training the system.

#### 2.4.2. System Model for Municipality Section Seasons

To further assess the efficiency of the given datasets we preprocessed row data and produced a new set of datasets for each municipality section. Preprocessing lead us to adopt the use of season to separate the initial datasets. Specifically, for each of the datasets presented in Table 1 and Table 2 we created 4 new datasets. Each of these datasets contain the context of each season, thus the number of assigned months per season is 3 months. Such a mutation allow us to build more robust classification models to support the adopted LSTM inference engine model. Since this is an optimization of the proposed classification models we applied such extension only to Papagos section and Cholargos section that have result in more efficient classification models than the whole municipality of Papagos–Cholargos dataset. Specifically, in Table 5 and Table 6 there are presented the modified section datasets for both municipality sections. Note that in Table 5 and Table 6 we present the features (i.e., attributes) with regards to certain types and values obtained for each of them, such features are used to input the proposed LSTM inference model.

### 2.5. Evaluation Method and Metric

#### 2.5.1. 10-Fold Cross-validation Evaluation Method

We evaluated the proposed LSTM classification model with 10-fold cross-validation evaluation method. Specifically, for each proposed system model dataset we created a set of training and testing datasets with the following procedure. For a certain loop of 10 repetitions we separated the initial dataset to 10 parts, where 9 parts were used to form a new training dataset and the remaining 1 part is used for testing dataset. Note that in each iteration, a certain part cannot be contained simultaneously both as a part of the training dataset and as a testing dataset. Thus, the training and testing datasets can only contain different records. When the training and testing datasets were created in each loop, they are feed to the classification model to assess its efficiency. This process is repeated until all the parts are used for training and testing.

#### 2.5.2. Prediction Accuracy Evaluation Metric

Efficiency of the classification is assessed by prediction accuracy quantitative evaluation metric, a∈[0,1] that is defined as presented in the following equation:

(1) $$a=\frac{t_{p}+t_{n}}{t_{p}+f_{p}+t_{n}+f_{n}}$$

Where, $t_{p}$, are the instances that are classified correct as positives and $t_{n}$, are the instances that are classified correct as negatives. In addition, $f_{p}$, are the instances that are classified false are positives and $f_{n}$, are the instances that are classified false as negatives. A low value of a means that the adopted classification model is weak while a high value of a indicates that the proposed classifier is efficient.

## 3. Results

### 3.1. Experimental Parameters

#### 3.1.1. Municipality Parameters

Experimental parameters are incorporated to perform certain experiments and observe subsequent results. The municipality parameters are a special case of experimental parameters that are important because they define the context of the experiments to be performed by the proposed system. Specifically, the whole area we are experimented is the municipality of Papagos–Cholargos located in Athens, Greece. The municipality of Papagos–Cholargos has a total coverage area of 7.325 square kilometers (Km^2^). The municipality is further divided into two neighboring sections, the section of Papagos and section of Cholargos. section of Papagos covers an area of 3.375 km^2^, while section of Cholargos covers an area of 3.95 km^2^. Population demographics of municipality and neighboring sections are important to have an in depth knowledge about the population that is supported by our system. The municipality of Papagos–Cholargos has in total 54,539 citizens, who are divided in 23,699 citizens living at the section of Papagos and 30,840 citizens living at the section of Cholargos. Table 7 summarizes municipality parameters.

#### 3.1.2. Datasets Parameters

Municipality parameters provide the context to work with the emerged daily municipality problems. Such problems need to be served by the proposed system. Since the occurred problems are time stamped and logged into the system they produce certain datasets with adequate size in records for a certain amount of time. Specifically, municipality of Papagos–Cholargos provided us real datasets through the Citify municipality software application that contain the number of emerging problem in a period of a year. In total the dataset that contains information about the whole municipality of Papagos–Cholargos has size 351,657 records. In addition, the section of Papagos has a dataset that is a subset of the total dataset and has size of 130,581 records. Consequently, the section of Cholargos also has a dataset of 221,076 records. These data are used by our system for training and testing of the adopted inference engine LSTM classification model. The provided datasets parameters are summarized in Table 8.

#### 3.1.3. LSTM Classifier Tuning Parameters

Inference engine LSTM classification model is built based on the provided datasets. We used an LSTM neural network implementation of the Weka workbench that is open source artificial intelligence software [6]. We used the application programming interface (API) and the GUI provided by Weka to tune LSTM neural network parameters. Experimental tuning of the LSTM classification system leads us to adopt certain tuning parameters, as also performed in [38], where an applied LSTM inference classifier is tuned according to the Weka API feasibility. Specifically, input layer is composed of 6 nodes (i.e., one for each of the six predictive attributes). Number of hidden layers is defined to be 3 layers. The first hidden layer has 32 nodes, second hidden layer has 64 nodes and third hidden layer has 32 nodes. The output layer has 10 nodes (i.e., one for each department class attribute). The batch window size to feed the LSTM neural network is 100 records. Learning rate is defined to be 0.001 while number of epochs are 10. Hidden layers’ activation function is ReLu, while output layer activation function is SoftMax. Table 9 summarizes the LSTM classification model tuning parameters.

### 3.2. Prediction Accuracy Classification Results

#### 3.2.1. Prediction Accuracy of Municipality and Municipality Sections per Year

**Figure 6:**
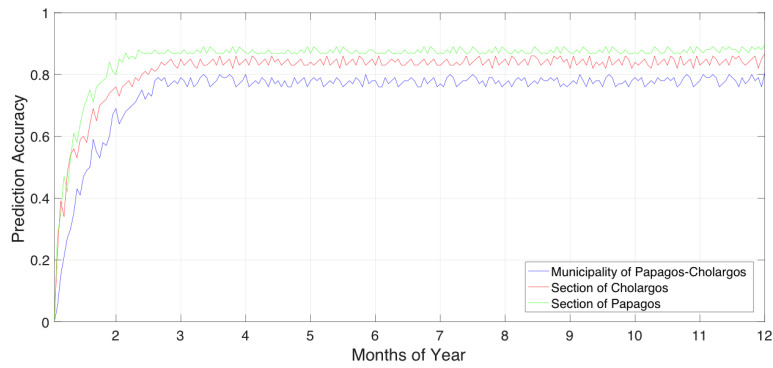
Prediction accuracy of municipality and municipality sections per year.

We used a 10-fold cross-validation to evaluate the proposed inference engine model, in which we observed certain values of prediction accuracy, a. The experiments are based on the given datasets assigned to the municipality of Papagos–Cholargos—as well as the sections of Cholargos and Papagos, respectively. We ran the experiments for the period of one year with real data provided by the Citify municipality software application. The prediction accuracy, a, indicates the ability of the LSTM neural network classifier to predict which department (i.e., municipality resource) will be allocated to serve a certain problem that will arise in the municipality coverage area. During this period, we observed a=0.8061 for the case the whole municipality of Papagos–Cholargos. Concretely, we can observe a=0.8678 for the section of Cholargos as well as a=0.8953 for the section of Papagos. The prediction accuracy, a, of the municipality of Papagos–Cholargos and the municipality sections Cholargos and Papagos, respectively, per year is presented in Figure 6.

#### 3.2.2. Prediction Accuracy of Municipality Sections per Season

**Figure 7:**
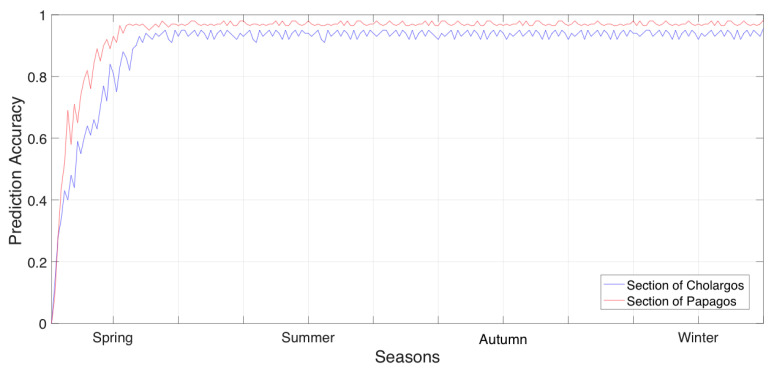
Prediction accuracy of municipality sections per season.

The same evaluation method of 10-fold cross-validation and prediction accuracy, a, evaluation metric is used to assess the efficiency of the proposed system in the case we treat sections of Cholargos and Papagos with certain preprocessed datasets that are enhanced with seasonal information. This is feasible due to the exploitation of local seasonal monthly information emerged by further processing of the initial datasets. Specifically, in the case of the Cholargos section, we can observe a=0.9551, while for Papagos section we observe a=0.9822. The prediction accuracy, a, of municipality sections Cholargos and Papagos, respectively, per season is presented in Figure 7.

### 3.3. Actual and Predicted Distribution of Municipality Section Resource Allocation per Season

**Figure 8:**
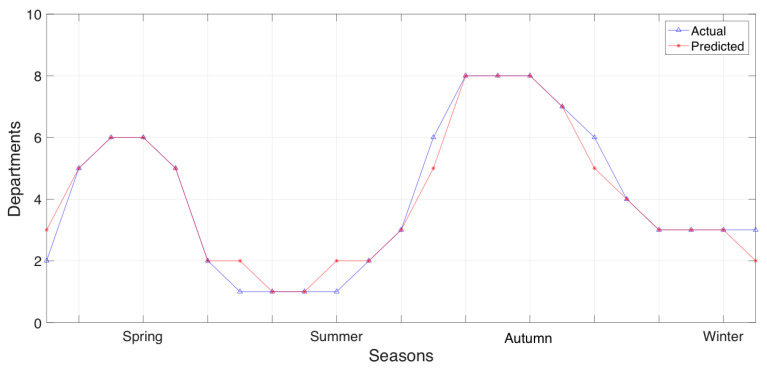
Actual and predicted distribution of municipality section Papagos department resource allocation per season.

Further experiments focused on the distribution of municipality sections’ department allocation that is treated as resource allocation, per season. First we experimented with the section of Papagos exploiting its prediction accuracy, a, to visualize the actual and predicted distribution of municipality department resource allocation per season. This information is presented in Figure 8.

**Figure 9:**
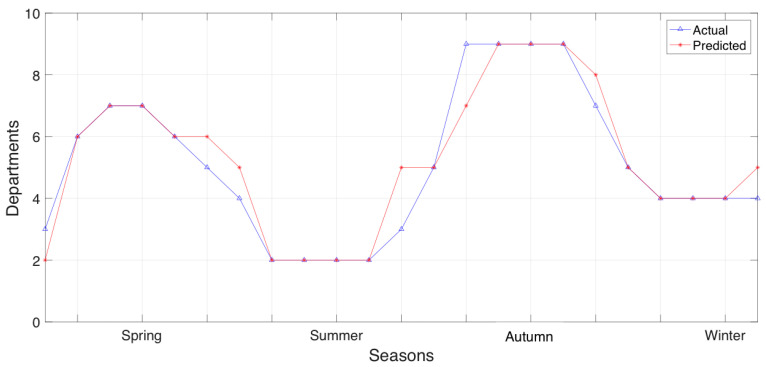
Actual and predicted distribution of municipality section Cholargos department resource allocation per season.

Consequently, we expanded our experiments to the section of Cholargos, exploiting prediction accuracy, a, to visualize the actual and predicted distribution of the municipality department (i.e., resource) allocation observed per seasonal data. The municipality department allocation for the section of Cholargos is presented in Figure 9.

## 4. Discussion

The current study conducts research in the area of municipality department’s resource allocation for citizens’ efficient wellbeing at the municipality of Papagos–Cholargos. Living in such a large municipality, within the smart city of Athens that has two separate sections (i.e., Papagos and Cholargos) covering a total area of 7.325 square kilometers and a population of 54,539 citizens is challenging with regards to everyday infrastructure problems that may arise during a period of a year. The occurrence of a problem triggers the municipality processes to face and handle it. We built a system that incorporates citizens’ feedback to assure a better quality of life. The proposed system exploits both the potentiality of crowdsensing and crowdsourcing paradigms acting at the edge of the infrastructure to track and annotate emerged problems. The rational that lead us to combine both paradigms is that crowdsourcing can elevates wisdom of the crowd to face a problematic situation while crowdsensing is able to track, annotate and submit the problem at the edge of the municipality infrastructure. In our research such paradigms complete each other to provide a viable smart city solution for the municipality. We used the Citify software application to cover the needs of the system’s actors (i.e., citizen and municipality control center).

Data sources and annotated upcoming problems submitted to the system are then used to train and test an LSTM neural network machine-learning model. Users act as voluntary human sensors that means that they track a problematic event in the municipality coverage area and then they annotate it through the Citify mobile application. Hence, users are raising the alert in the first place. Time stamped annotated data provided by users is used to extract features and feed the LSTM model. Specifically, such data contain all the information needed to input the system according the feature description provided in Table 1, Table 2, Table 3, Table 4, Table 5 and Table 6. Images are not analyzed, but just used to report the event type. However, reported event type is used as input to system as described in Table 4. In addition, spatial data are decomposed to certain road addresses within the municipality area as described in the appropriate feature of road address data attribute in Table 1, Table 2, Table 3, Table 4, Table 5 and Table 6. Such a model is trained on real data for a period of a year. Data are preprocessed to discard repeated or missing values to assure that each part of the dataset has impact in the decision making of the inference model. We experimented with certain parameters, such as the whole municipality dataset or two separate sections’ datasets. We also experimented with the whole year or seasonal parts of the year. We evaluated our model with certain evaluation method and metric, such as 10-fold cross-validation and prediction accuracy of an examined model. We found that better results are observed by assuming data sources in the form of seasonal data, since this makes the datasets less complex. We also fine-tuned the API of the LSTM neural network model that is available by Weka machine-learning workbench, to provide a better model. Applying our assumptions and available data sources to the fine-tuned model we observed certain results that prove that the incorporation of our model is essential in facing emergencies in the municipality by assigning certain problem to the available department resource for further processing and solving it.

We are currently using the LSTM model as the inference model of the proposed system. We do not perform pure research in the area of LSTM, but rather, we use its potentiality to apply it to our problem. This is why we tuned the LSTM model with the provided Weka API. However, we describe extensively (i.e., in Section 3.1.3) the proposed network structure (i.e., input, hidden and output layers) as well as the appropriate activation functions and other parameters. Since it used in the area of applied research we perform fine tuning given the options the API was able to provide us. Such information can be used to rebuild the classifier and reproduce the results we obtained providing certain input values from the municipality of Papagos–Cholargos.

Analyzing the results obtained during the experimentation process, we can observe certain trends. In Figure 6, we can see that the prediction accuracy of the Papagos–Cholargos municipality is less than the prediction accuracies gained by the two separate municipality sections of Papagos and Cholargos, respectively. This is explained as follows: since the Papagos–Cholargos municipality dataset, during a year, is more complex since it covers more spatial area than each of the two sections individually. The more complex the relation in a dataset the less prediction accuracy is observed. In addition, such complexity of the datasets lead to a higher prediction accuracy of Papagos municipality sections than a lesser prediction accuracy of Cholargos municipality section since section of Papagos has less coverage area than section of Cholargos. Due to this fact we continue to experiment with the datasets of the two sections in the next experiments.

The results in Figure 7 are promising in adopting fewer complex datasets that cover separate seasons since with this technique datasets are becoming even more compact thus less complex. In this case it also holds that the dataset of Papagos section has higher prediction accuracy than the dataset of Cholargos section. We can observe that, in the case of seasonal datasets, both classifiers are converging to the higher values of their prediction accuracy earlier than they converge in the case of yearly datasets. This is also explained due to the simplicity of the seasonal datasets compared with the yearly datasets.

In Figure 8, we explain the results observed in Figure 7 with regards to the number of the predicted departments per season that will used to serve an upcoming future event in the municipality section of Papagos. Comparing Figure 8 with Figure 9, we can observe that the section of Papagos can predict in greater detail the municipality department allocation that it does in the section of Cholargos. Currently, the results of department resource allocation provided in Figure 8 and Figure 9 can explain the prediction accuracies observed in Figure 7, since in all these cases, the datasets are based on seasonal information.

The experimental setup conducted in the proposed research is not naively simple, but rather a well-defined process. We experimented with three different types of datasets that feed the LSTM inference model with different predictive attributes. Specifically, the first case contained datasets of both Papagos and Cholargos municipality sections, where the original municipality dataset is divided according to the territories of each municipality section—thus forming a derivative approach. The second case contained the whole dataset of the municipality of Papagos–Cholargos and experiments with it holistically. We performed experiments with the datasets provided by the first and second experimental setups and observed better results in the case of considering two different datasets (i.e., one for each municipality section). At this stage, we enhanced the prediction accuracy of the model by adopting a third case of experiment that evaluated the system based on the datasets of each municipality sections of Papagos and Cholargos with the adoption of the predictive attribute of seasonal information. It is proved that with this approach we have more efficient results compared to the two previous approaches. The evaluation method we incorporated is 10-fold cross-validation and is capable to handle emerged events that do not belong to the training dataset since the emerged events are always infrequent, but also have stochastic nature in the context of our approach. For achieving that, 10-fold cross-validation uses nine parts of input data as training data and the remaining one part as test set in a 10-fold loop, so as to prevent over fitting of the LSMT model. In addition, we clarify that there are not the users that make the prediction or the evaluation; instead, it is the system itself. Specifically, the system makes a prediction for the future ex-ante and the accuracy of the system is evaluated ex-post by observing the actual situation in the future. Hence, prediction accuracy in this context is suitable and convenient to assess efficiency of the proposed system. Please also note that there is not user intervene in the middle of the process, Instead, it is a machine-learning intelligent inference model that makes such mapping possible.

The proposed system is based on artificial intelligence methodology, methods and techniques. In this context, the efficiency of the adopted LSTM deep-learning model is not assessed through descriptive statistics measures and metrics such as the statistical average. Instead, it used for the widely accepted predictive statistics metric of prediction accuracy to evaluate the efficiency of the proposed model. So, such a comparison is out of the context with the current methodological concept. Currently, throughout the study we have presented and analyzed the adopted application interfaces as well as we have added a separate subsection that examines in great detail the system architecture as the core of system overview. In that section we present the back end intelligence of the system as well as the logic flowchart of the adopted process in which the system is based. The introduction of the emergency manual is considered as the outcome of this study since it is able to provide stochastic resource allocation. Stochastic knowledge has implicitly the definition of the ex-ante and ex-post intuition provided by certain rules that make the matching happen in reality. Such rules are the outcome of the proposed LSTM model. However, since LSTM is a special case of deep learning the resulting model and produced rules cannot interpreted by humans, since this is a NP hard problem. However, humans can evaluate empirically the effectiveness of the proposed model and implied rules through to prediction accuracy evaluation metric. The experimental part is defining the artificial intelligence methodology it is followed to draw certain results. During experiments, it used all the available dataset to evaluate the adopted model. Specifically, it used 10-fold cross-validation as the evaluation method. This does not mean that it used only 1/10 of the available data. Instead, it used all the data with a round loop of 10 steps where for each step the evaluation method uses the 9/10 of the data for training and the remaining 1/10 for testing. Please note that at the end of the round loop all the data were used either to train or to test the model, but at each time data are separated to make sure that not a single data are used for both training and testing at the same loop.

The exact number of user requests is out of the scope of the current study. This is because we are not interested in the exact number of the users and the frequency at which they are alerting the system and adding data to the knowledge base. We make the assumption that we already have the knowledge base regardless of how exactly it was created that is the truth in our study. Qualitatively, we know that there are a certain number of users that were involved in the creation of the knowledge base, but we have no access to this information, as we are external researchers and not employees of the municipality. Thus, we assume that this number of users could be from one to many users—even the whole population of the municipality. However, we are not interested in this part, as it has nothing to do with the efficiency of the system or the observed prediction accuracy as designed and described in this study. Specifically, we are interested in what is happening after users trigger the proposed model that means that the users are voluntarily taking part on this process—without any external incentive—since they are satisfied to be able to make their municipality a green ecosystem for somebody to live and wellbeing. The use of the Citify app is conceptually located in the frontiers of the proposed system (i.e., an interface with the system part of the environment), since we do not have any in depth knowledge of how it is built as an autonomous system. Instead, we use Citify app as a system application that has two interfaces: one for the users, and one for the municipality. Such context of the Citify app is explained in great detail in the appropriate sections. Please note we do not go deeper into analyzing the Citify app since it is considered an interface tool and not the heart of the artificial intelligence model. Currently, we focus on the municipality of Papagos–Cholargos since it is providing the energy and resources of our experimental setup. Throughout the study, we discuss the methods, materials, participants in great detail, and discuss the results found in the current study.

This is a new system that is being developed for use by the municipality of Papagos–Cholargos to provide the conditions of a green ecosystem used by its citizens. The system is not commercially available, as in its current form, it is a pure research artifact that is new and being developed by the researchers. The next step is to promote such a system for commercial use by the municipality and appropriate collaborative vendors. Currently, the proposed system cannot be compared equally with traditional solutions like linear mapping or averaging, since such approaches are—by definition—considered monolithic, compared with a deep learning LSTM model. To make it clearer, LSTM is able to capture nonlinear relationships emerged by the observed data that make it a more robust model than it is linear mapping and averaging. Currently, this is the main difference between the static nature of descriptive statistics and the dynamic nature of predictive statistics. In addition—even in the area of predictive statistics—the LSTM deep learning model is a state-of-the-art model, outperforming other predictive statistics models for making predictions exploiting nonlinear nature of input data sources.

## 5. Conclusions

We treat municipality department resource allocation problem as a LSTM stochastic prediction process. Citizens are empowered with smartphones and act as human sensors to track, annotate and report malfunctions in the municipality coverage area. The municipality of Papagos–Cholargos is divided into two sections for better handling of emerging events and assigning them to the appropriate departments. Since this is a stochastic process, data collected through environmental crowdsourcing and smartphone crowdsensing processes at the edge of the municipality infrastructure can be input to an inference engine classification model. It has been shown that the division of the municipality into two sections—and the further analyses of data to seasonal datasets—leads the adopted system to be more effective than using the whole municipality and yearly use datasets. In the future, we aim to penetrate more in the municipality physical infrastructure and be able to assign emerging problems at the granularity of employees per department for better supporting the operational process of the municipality of Papagos–Cholargos.

## Figures and Tables

**Table 1:** Papagos municipality section dataset description.

Attribute	Type	Values
Road address	Predictive nominal	[1…10,000]
Problem	Predictive nominal	[1…15]
Month	Predictive nominal	[1…12]
Sector	Predictive nominal	[1…20]
Department	Class nominal	[1…10]

**Table 2:** Cholargos municipality section dataset description.

Attribute	Type	Values
Road address	Predictive nominal	[1…12,000]
Problem	Predictive nominal	[1…15]
Month	Predictive nominal	[1…12]
Sector	Predictive nominal	[1…24]
Department	Class nominal	[1…10]

**Table 3:** **Municipality** of Papagos–Cholargos dataset description.

Attribute	Type	Values
section	Predictive nominal	[1,2]
Road address	Predictive nominal	[1…22,000]
Problem	Predictive nominal	[1…15]
Month	Predictive nominal	[1…12]
Sector	Predictive nominal	[1…44]
Department	Class nominal	[1…10]

**Table 4:** Attribute problem value description.

Description	Value
Abandoned vehicle	1
Stray animals	2
Broken sign	3
Broken pavement	4
Street light problem	5
Drainage problem	6
Pothole	7
Water leak	8
Graffiti on wall	9
Illegal trash	10
Illegal parking	11
Parking on wheelchair ramp	12
Dead animal	13
Damaged bench	14
Bicycle road problem	15

**Table 5:** Papagos municipality section per season dataset description.

Attribute	Type	Values
Road address	Predictive nominal	[1…10,000]
Problem	Predictive nominal	[1…16]
Month	Predictive nominal	[1…3]
Sector	Predictive nominal	[1…20]
Department	Class nominal	[1…10]

**Table 6:** Cholargos municipality section per season dataset description.

Attribute	Type	Values
Road address	Predictive nominal	[1…12,000]
Problem	Predictive nominal	[1…16]
Month	Predictive nominal	[1…3]
Sector	Predictive nominal	[1…24]
Department	Class nominal	[1…10]

**Table 7:** Municipality parameters.

Parameter	Coverage Area in km^2^	Population
Municipality of Papagos–Cholargos	7.325	54,539
Municipality section Papagos	3.375	23,699
Municipality section Cholargos	3.95	30,840

**Table 8:** Dataset parameters.

Dataset	Dataset Size in Records
Municipality of Papagos–Cholargos dataset	351,657
Municipality section Papagos dataset	130,581
Municipality section Cholargos dataset	221,076

**Table 9:** Long short-term memory (LSTM) classifier tuning parameters.

Tuning Parameter	Value
Input layer	6 nodes
Number of hidden layers	3
1st hidden layer	32 nodes
2nd hidden layer	64 nodes
3rd hidden layer	32 nodes
Output layer	10 nodes
Batch window size	100 records
Learning rate	0.001
Number of epochs	10
Hidden layer activation function	ReLu
Output layer activation function	SoftMax
